# Collectanea: Miscellaneous

**Published:** 1834-07-01

**Authors:** 


### MISCELLANEOUS.

### KING JAMES'S COUNTERBLASTE TO TOBACCO.

In the reign of James the First the habit of smoking was so universal in England, that a large proportion of his Majesty's liege subjects had become little better than walking volcanos; whereupon that learned and facetious monarch, distressed at the prevalence of so outlandish a custom, was at the trouble of writing a "Counterblaste to Tobacco," in which he exposes the turpitude, as well as the insalubrity of this horrible practice. He shews the baseness of its origin in the use made of it by the Indians for the cure of a certain disreputable malady, "whereunto these barbarous people are (as all men know) very much subject, what through the uncleanly and adust constitution of their bodies, and what through the intemperate heat of their climate; so that as from them was first brought into Christendome that most detestable disease, so from them likewise was brought this use of tobacco, as a striking and unsavourie antidote for so corrupted and execrable a maladie; the stinking suffumigation whereof they yet use against that disease, making so one canker or venim eate out another."

"Now to the corrupted baseness of the first use of this tobacco doeth very well agree the foolish and groundlesse first entry thereof into this kingdome. It is not so long since the first entry of this abuse amongst us here, as this present age cannot yet very well remember both the first author and the forme of the first introduction of it amongst us. It was neither brought in by king, great conqueror, nor learned doctour of physicke. With the report of a great discovery for a conqueste, some two or three savage men were brought in, together with this savage custom. But the pitie is, the poore wild barbarous men died, but that vile barbarous custom is yet alive, yea in fresh vigor."

His majesty introduces some original physiological and pathological views. The operation of the noxious weed as a sialogogue is thus illustrated, in opposition to those who maintain that it acts beneficially by purging the head and stomach of rheums and distillations: "The fallacie of this argument may easily appeare by my late preceding description of the meteors; for, even as the smoakie vapours sucked up by the sunne, and stayed in the lowest and cold region of the aire, are there contracted into clouds, and turned into raine and such other watery meteors, so this stinking smoake, being sucked up by the nose, and imprisoned in the cold and moyst brains, is, by their cold and wet facultie, turned and cast forth againe in watery distillations, and so are you made free and purged of nothing but that wherewith you willfully burdened yourselves."

The post-mortem appearances in inveterate cases of fuliginous disease are duly recorded.

"Surely smoke becomes a kitchen farre better than a dining chamber, and yet it makes a kitchen also oftentimes in the inward parts of men, soyling and infecting them with an unctuous and oily kind of soote, as hath been found in some great tobacco-takers that after their death were opened." A state obviously irremediable, unless Archeus, in the multiplicity of his functions, should assume that of a chimney-sweep.

The blast of the royal horn winds up in the following energetic expostulation:

"Have you not reason then to be ashamed, and to forbeare this filthie noveltie, so basely grounded, so foolishly received, and so grossly mistaken in the right use thereof? In your abuse thereof sinning against God, harming yourselves both in persons and goods, and raking also thereby the markes and notes of vanitie upon you; by the custom thereof making yourselves to be wondered at by all forreine civill nations, and by all strangers that come among you to be scorned and contemned; a custome loathsome to the eye, hateful to the nose, harmefull to the braine, dangerous to the lungs, and, in the blacke stinking fume thereof, nearest resembling the horrible Stigian smoake of the pit that is bottomless."

Notwithstanding all this, we strongly suspect that if the worthy monarch could once have been brought fairly under the influence of a good cigar, he would have found it so congenial to his lazy and contemplative disposition, that he would have revoked the Counterblaste and written a Currus Triumphalis è Tabacco.

### STATE OF DRUGS IN LONDON.

In our administration of medicine, we expect and assume an identity of operation, from things widely different in their elementary nature and composition. There is, I was assured only the other day, by a very intelligent chemist and druggist, a difference of 300 per cent. in the quality of certain articles of the Pharmacopœia, on sale in different shops. This was his own unreserved expression; and the statement may be verified by any one who will take the trouble to examine samples from various retail stores in the city of London. Indeed, this difference in the quality of certain drugs may be at once assumed, from the difference of prices in the bills of parcels from various wholesale houses. I have known this difference range from 12s. to 20s., from 1s. 8d. to 6s.; and this in medicines declared to be of the best quality, and furnished to a great public institution, where it was known that they would be submitted to a fair competition, and would be carefully examined by a competent professional committee. It is not likely that better drugs than the worst of these should be furnished to the solitary practitioner of the retired hamlet. Now, even admitting a range in what may be termed the fancy price of drugs, as measured by an imaginary excellence of colour and other properties of appearance, there still would be an alarming difference in their money value, only to be accounted for by a difference in their useful medicinal properties. By all great dealers this variety is admitted; and yet, be it remembered, every variety finds a purchaser. A great deal of this pernicious trash is disposed of to the London market; but the greater part of it, I am told, "goes into the country." The respectability of the drug trade has been greatly lowered by the demand of the retail dealers for these low-priced inferior samples. I have heard this stated by respectable druggists, who have lamented that it should be so. Those who conduct the trade with a sufficient capital, a capital enabling them to make a selection in the market, and who are impressed with the responsibility of their business, would, I am sure, be very glad to assist in placing it on a footing, which, by affording more security to the public, would imply its greater respectability. There are some houses where wonderful pains are taken to insure excellence in the medicines furnished, and accuracy in the preparation of them. Like every thing in London that is fairly taken up, the business in these houses is done to perfection. Of all the numerous trades and corporations of our citizens, there is, indeed, no one more intelligent, and in every way respectable, than the better, I mean by this the scientific, class of our druggists.

I will not enlarge on the mischief that must ensue to the public from the circulation of so much bad physic; the nuisance is gross and palpable; the simple expression of it is its strongest condemnation; and it does not attach to me more than to others to exclaim against it; but I may be permitted to express my sincere regret at the uncertainty in which it involves the science of medicine, and the discredit which it brings on its practice. In prescription no one now knows what he orders. How, then, can he presume to calculate upon the effects of his prescriptions, or to reason on them when obtained? From this constant source of error, I find it impossible to procure any information as to the effects of varied doses of medicine that can be at all depended on.

In the very outset of the Materia Medica, in the study of its elements, we are met by this perplexity, arising from the abuses of the drug trade. There is another bar to our improvement in the knowledge of drugs, as connected with the sale of them, which is the way in which they are dispensed. The materials may be of good average quality, yet may be compounded in wrong proportions, or by a process of manipulation inconsistent with the intentions of the prescriber. From the general ignorance among medical men on the subject of drugs, the prescriber may often mistake his medicines and their doses; he may be, and he often is, careless, or by chance inaccurate, but securities are taken from the Physician by age and education, to prevent, at all events to limit, his means of injuring the public. The compounder of his prescription is in most instances amenable to no inquiry, to no tribunal. In a shop that has been mentioned to me, a common porter has been habitually employed to compound medicines; and I have recently met with an instance in which the entire business was carried on, for months together, by a widow, with an infant in her arms. Boys and women have not unfrequently, in my own observation, been found in charge of a shop and its business during the absence of "the master."

The consequences are best illustrated by the police reports of the daily papers, and by such advertisements as the following: "The gentleman who purchased the carbonate of soda, tartaric acid, and sp. lavendulæ comp. is requested not to use them, but to apply for information as soon as possible."—See Morning Herald, Sept. 8, 1829. Some of those now present may remember the attempts which I made, in my lectures of last year, to direct the attention of the College to the disgraceful uncertainty of the preparations of Colchicum in the different shops of this metropolis. In some the "Vinum Colchici" is a wine of the fresh root; in some a spirit of the dried root, or a spirit of the fresh root, and a wine of the dried; a wine of the seeds, or spirit of the seeds; varying again in the quantity of the ingredients, and in the time given to the preparation. In no two shops, visited consecutively, would the Vinum Colchici, as ordered by the Pharmacopœia, be met with; and certainly in no two would it be found to possess the same degree of strength.

In a tour of visits made officially to the retail shops, it was found that the "Cathartic Extract" was hard and black in all places but one, where it was soft and mouldy, being "seldom asked for." A proprietor of Senna Confection candidly acknowledged, that the light brown, dirty-looking compound, was not fit for "human medicine, but it might be worked up into boluses for horses." Oxalic acid was generally met with in a large bottle, on the lower shelf, and in front of the shop, next to sulphate of zinc, and other salts. White arsenic was in most instances kept as a powder. It was seldom marked "poison," and it did not appear that any precaution was observed in its sale. In more than one instance I found this deadly agent in a bottle bearing two different labels on its opposite sides.—*Med. Gazette.*

### MR. RICHMOND'S MUSICAL PERFORMANCE.

The effects produced are the following: two series of sounds are heard; one exactly resembling the notes of a musical snuffbox, the other a succession of bass sounds; or a drone, which necessarily goes along with the higher notes.

Of the two series of notes, the first, or those which resemble the tones of a musical snuffbox, are not produced in the larynx; the second or the bass notes, on the contrary, are laryngeal. Mr. Mayo ascertained these points by the following observations. It is well known, that when sound is produced in the larynx, on its becoming grave the larynx always sinks in the throat; on its becoming sharper, the larynx always rises. But in Mr. Richmond's performance the larynx does not change its position with the alteration of the higher series of notes; while, on the contrary, it manifestly rises and falls with the alterations of the bass sound.

The bass sound then is laryngeal; but how are the higher tones produced?

The principle was originally discovered by Mr. Wheatstone, in analyzing the production of the musical sounds of the Jew's-harp, and by him shewn to apply to Mr. Richmond's performance. It is this.

Different volumes of air, like strings of different lengths, vibrate with different frequencies, and produce therefore different notes. Now if a vibrating tongue, such as that of a Jew's-harp, be made to vibrate before a volume of air, the vibrations of which are of the same frequency, the sound of the musical tongue causes the volume of air to vibrate, and two sounds in unison are produced. But suppose the volume of air not to be unisonant with the tongue of the Jew's-harp, in some cases the volume of air will speak, or be thrown into vibration, in others it will not. A second sound will be produced, Mr. Wheatstone discovered, when the volume of air is one which vibrates multiples of the sound of the musical tongue. If, for instance, the tongue of the Jew's-harp vibrate 32 times in a second, volumes of air, that vibrate 64, 96, 128 times in a second, will be thrown into vibration through its vibrations. The skill of the performer on the Jew's-harp consists in shaping the cavity of the mouth so as to hold in succession such definite volumes of air as, being presented to the vibrating tongue of the instrument, reciprocate different multiples of the bass sound it produces.

These multiples are not found in sufficient number to contribute to the performance of a melody till the third octave above the bass sound, nor without the use of more bass sounds than one. Mr. Eulenstein, in his performance on the Jew's-harp, uses six instruments, rapidly substituting one for another, as he wishes to use multiples of a different bass sound.

In Mr. Richmond's performance the larynx is equivalent to the Jew's-harp in Mr. Eulenstein's, but, with this advantage in favor of the former, that it is much easier to alter the tension of the vocal chords in the larynx than to lay down and take up the Jew's-harp. The rest of the performance is exactly the same. The cavity of the mouth is shaped to reciprocate multiples of the bass notes, in a succession which forms a musical air. H. M.

### THEORY OF ANIMAL MAGNETISM.

I shall conclude this paper with the following observations: For some time past, our physiological theories have manifested a decided tendency towards materialism. Of late, indeed, we seem to have got so much into the habit of speaking of the mental functions as being performed by certain organs, that we would appear to have entirely forgotten that every organism requires to be vivified by an active principle, that the employment of an instrument presupposes the existence of an intelligent agent. In our speculations upon these matters, we become so much interested in the play of the puppets, that we totally overlook the moving power; while contemplating the conditions of intelligence, we become blind to the principle.

But sensation and thought, as has been frequently remarked, are neither the properties nor any of the necessary effects of matter; material structure alone cannot be the cause of the vital phenomena; it may supply the organs or tools through the medium of which these are ostensibly manifested, but these manifestations cannot take place without the operation of an intelligent cause. "That there is some invisible agent in every living organized system, seems to be an inference to which we are led almost irresistibly. When we see an animal starting from its sleep, contrary to the known laws of gravitation, without an external or elastic impulse, without the appearance of electricity, galvanism, magnetism, or chemical attraction; when we see it afterwards moving its limbs in various directions, with different degrees of force and velocity, sometimes suspending and sometimes renewing the same motions, at the sound of a word or the sight of a shadow, can we refrain a moment from thinking that the cause of these phenomena is internal, that it is something different from the body, and that the several bodily organs are nothing more than the mere instruments which it employs in its operations?"*

In the human economy, this invisible agent, this intelligent principle, which operates through the medium of certain corporeal organs, is called the soul. In the normal state of the organism, we know that our faculties of sensation and perception, at least, are exercised through the instrumentality of certain material organs, and hence physiologists have been led to consider these instruments as the necessary conditions of the exercise of these faculties. But are we prepared to maintain that, in any circumstances, the soul is incapable of exerting its energies in a different manner? Do we hold that it is the eye alone that sees, the ear alone that hears, &c. or shall we admit that there is an internal sense to which the impressions of sight, hearing, &c. are conveyed, and to which the material organs are merely subservient? And if we are disposed to make this admission, can we deny the possibility of impressions being communicated to this internal sense, in some extraordinary manner, without the necessary intervention of the usual organs? This is a question which is capable of being solved by experience; and if the cases I have adduced in this paper have been accurately observed and faithfully reported, of which there seems no reason to doubt, the question may be considered as having been already satisfactorily decided in the affirmative. If the phenomena observed are calculated to excite our wonder, and to call forth our scepticism, if they appear to be inexplicable and irreconcilable with any of our previous notions, let us remember that the cause of this may be found in the narrowness and imperfection of our preconceived systems; and this consideration should lead us to a careful review of the principles of our knowledge, rather than to an obstinate and irrational denial of the facts presented to us by experience.

Nil adeo magnum, nec tam mirabile quidquam,
Quod non paullatim minuant mirarier omnes.
Desine quapropter, novitate exterritus ipsa,
Expuere ex animo rationem; sed magis acri
Judicio perpende: et, si tibi vera videntur,
Dede manus; aut, si falsum est, adcingere contra.—LUCRETIUS.

—*Colquhoun on Animal Magnetism.*

* Barclay, on Life and Organization, p. 370.

STATE MEDICINE IN GERMANY.

*Proceedings of the Practical School of State Medicine in the University of Berlin, during the Summer Semester of* 1833. *By* Dr. Wagner, *Professor and Stadt-Physikus.*

The number of students who attended this course amounted to thirty-four; among whom were several doctors of physic and other licensed practitioners, most of them anxious to prepare themselves practically for the physicate examination. The number of cases submitted to us for medico-legal inquiry was 129, almost every one of them adapted for the instruction of the class. Reports, written out and argued, some of them in much detail, were severally drawn up on those cases.

A good deal of other business also was allotted to the students: for example, they had the opportunity of opening many bodies which did not absolutely require forensic investigation; these were the bodies of suicides, drowned persons, and of individuals who had been poisoned with sulphuric acid, &c. The chemical research was conducted in the latter cases, as well as in some inquiries connected with medical police, the adulteration of articles of food and medicine, for instance, by Dr. Gusserow: to him, and to Mr. Baerwald, the apothecary, who favoured us with the use of his laboratory, we have to acknowledge ourselves deeply indebted.

Of the 129 medico-legal cases, 113 related to living people, fifteen to persons found dead, and there was one case of poisoning with arsenic. Of the investigations on the living, ninety-four were concerning bodily injuries or ailments, and nineteen had reference to the state of the mind. Among the former, we were called upon to determine whether certain individuals who were arrested for debt, were able to endure the severity of imprisonment? whether others were fit to be brought up for trial, or to be removed from their homes? and whether certain persons were well enough to perform the duties of their respective offices? &c. Several of the alleged ailments in these cases turned out to be factitious, either altogether feigned, or whatever was really amiss was grossly aggravated: most of them were absurd complaints about chest affections, pains in the limbs, headachs, and piles. Where positive bodily injury had been inflicted, we were required to give our decision whether it came under that class denominated by the law injuries of a grievous nature, or whether (within the meaning of the criminal code) it involved a hazard of life, or was likely to entail a permanent mischief on the constitution or the limbs.

Among the remarkable cases of this kind which came before us, was the following one of attempted abortion. A young woman, seven months gone with child, had employed savine and other drugs, with a view to produce a miscarriage. As these had not the desired effect, a strong leather strap (the thong of a skate) was tightly bound round her body. This too availing nothing, her paramour (according to his own confession) knelt upon her, and compressed the abdomen with all his strength; yet neither did this effect the desired object. The man now trampled on the girl's person while she lay on her back; and as this also failed, he took a sharp-pointed pair of scissors, and proceeded to perforate the uterus through the vagina. Much pain and hæmorrhage ensued, but did not last long. The woman's health did not suffer in the least, and pretty much about the regular time a living child was brought into the world, without any marks of external injury upon it. It died indeed four days afterwards, but its death could not be traced to the violence inflicted on the mother's person: all the internal organs appeared normal and healthy.

Three cases of violation (stuprum puellæ immaturæ) were submitted to us. The subjects were children of from three to eleven years of age. Positive signs of actual violation could not be made out, but that the attempts had been made was quite clear. In one case, a venereal infection had been communicated.

In a couple of divorce cases, the husband was in one instance charged with having a venereal taint in his constitution, but it could not be proved; and in the other, the wife, who was already advanced in years, was ascertained to be unfit for one of the ends of matrimony, from peculiar malformation, or want of development of the sexual organs.

Among the cases which involved inquiries into the state of the mental faculties, fifteen were of a civil, and the other four of a criminal nature. The former related chiefly to the ability of managing property, and the removal of guardianship where recovery was alleged to have taken place. In one of these instances the individual was deaf and dumb. The criminal inquiries were concerning persons who had usurped nobility, resisted the lawful authorities, or committed homicide under circumstances which made it questionable whether they were responsible agents.

In our medico-legal examinations of the dead, we met with two cases which had terminated fatally in consequence of fractures of the skull, with extravasation of blood into the substance of the brain. In another instance, several injuries had been inflicted on the integuments of the head by a blunt instrument, but without any apparent mischief to the bones of the cranium. The parts got well in the course of a few weeks, and no disturbance of the intellectual faculties had been observed. But all of a sudden the patient became comatose, and died in a few days. On examining the cavity of the head, there was not only found a deposition of pus between the skull and the dura mater, but beneath the latter such a quantity of purulent fluid effused, that the left hemisphere was completely compressed by it, and reduced to an ash-coloured substance; but the right hemisphere and cerebellum were in a normal state.

In a child that had been run over and killed, the spleen and left kidney were crushed, and in consequence much blood had been effused into the abdomen and cellular substance about the kidney.

No external injury, however, except a very slight excoriation, was observable on the body.

We met with but a single case of infanticide; yet that one served to afford us ample opportunity for examining the various tests employed in those investigations.

One of the students of the class lost his life through a wound received in dissection, whereby the great vessels of the forearm, near the elbow, were fatally injured.

Several questions were investigated relative to the causes and consequences of alleged lesions, and with a view to determine whether certain wounds had been absolutely, or only contingently mortal. These inquiries involved many complicated considerations, a somewhat detailed account of which Dr. W. gives.

We were also called upon medico-legally to examine a human skeleton, which was found in a tolerably perfect state in digging up the foundations of a building. It belonged to a male subject of about the age of fourteen. No injuries could be detected upon it. But the point to be ascertained, if possible, was, whether this skeleton had lain in the earth above twenty years or not, as this was material with reference to its affording the corpus delicti for a criminal proceeding. An accurate scrutiny of the state of the parts, with especial regard to the nature of the changes which subjects undergo when interred in different soils, gave us reason to infer that this skeleton had been buried perhaps about twelve or fifteen years in the spot where it was found.

The following case of suicide was remarkable. A criminal, who had been shut up alone in a dark dungeon, when visited by his keeper, not long after, was found lying dead on the floor. It was thought at first that he had had a fit of apoplexy. A vein was opened, and other means of recovering him were tried, but to no purpose. It was now for the first time noticed that he had a foreign body in his mouth; and, upon examination, it proved to be a piece of woollen cloth two ells long and a quarter broad, a shawl, in fact, which the wretched man had thrust into his throat. Had this person been found dead under other circumstances, what reason would there not have been for suspecting that he had been murdered by a strange hand!

A case or two of malapraxis also came before us, particularly one in which, through the bad management of a midwife and an accoucheur, a woman had been allowed to sink after delivery, from uterine hæmorrhage.

And lastly, we investigated a solitary case of poisoning, in which a child had drunk from a cup that contained fly-poison (a preparation of arsenic.) Death ensued in twelve hours. But, notwithstanding the most minute search of the intestinal canal and its contents, not a vestige of the deleterious substance could be detected. This was most probably owing to the very small quantity taken, and the evacuation effected by the vomiting.—*Dr. Cummin, in Med. Gazette.*

### JOHN HUNTER.

In conclusion, gentlemen, let me express to you my conviction, that, as a physiologist and surgeon, John Hunter has had no equal in any age or country; that he was one of those powerful minds, appearing only at long intervals, of which this island, small as it is, has produced so great a number; that his name must be inscribed on that bright constellation of genius which already bears those of Harvey and Sydenham, of Bacon, Locke, and Newton, of Shakspeare, Milton, Scott and Byron. These gifted mortals, with kindred spirits, who have drawn inspiration from their example and works, shed over our land an intellectual glory equal to its renown in arts and in arms. The bosom of every Englishman glows with an emotion of conscious pride at the enumeration of these revered names. If, gentlemen, the time should ever come, when the institutions and the power of our beloved country shall have passed away, their memory would linger round the spots consecrated by their earthly labours; the land on which they trod would still be a watchword to the earth;—it would be peopled with the glorious recollections of its departed sages, as the sight of Greece recalled to the truly noble poet, who yielded up his life on her classic soil, the heroes who had fallen in her defence:

> "They fell, (he says,) devoted but undying;
> The very gale their names seemed sighing:
> The waters murmured of their name;
> The woods were peopled with their fame;
> The silent pillar, lone and gray,
> Claimed kindred with their sacred clay;
> Their spirit wrapped the dusky mountain,
> Their memory sparkled o'er the fountain,
> The meanest rill, the mightiest river,
> Rolled mingling with their fame for ever."

—*Lawrence's Hunterian Oration.*

### SYMPTOMS OF PAIN IN THE HORSE.

Pain is a symptom of most diseases; one that originates in disturbance or impression of the nervous textures distributed over the body. Speechless as the horse is, yet, as he is well known to possess many ways of making his wants understood in health, so in numerous instances under disease does he (to those acquainted with his ways) point out the seat, the kind, and the intensity of his sufferings, with an instinctive sagacity hardly to be credited by persons who are strangers to his manners and habits. The drooping, dolorous, desponding eye, in several chronic disorders; the wistful looking back at the flank, in pneumonia; the fearful, and even supernatural vividness of the eye in colic, just as the approach of another paroxysm is felt; together with the wild phrenzied roll of it, in times of violent pain and delirium; are so many examples of morbid physiognomy not to be misinterpreted by the practitioner of experience. Thus we may apply to horses what Sir Thomas Brown has said of men: "In long observation we may acquire a physiognomical intuitive knowledge, judge of the interiors by the outside, and raise conjectures at first sight."

[From *Hippopathology, a Systematic Treatise on the Disorders and Lamenesses of the Horse*, by Mr. Wm. Percival. It would be absurd in us to speak *ex cathedrâ* of this work; for, to confess the truth, we never doctored a horse in our lives; we must, consequently, leave the ἱππιατροι to give a detailed account of its merits. There is one point, however, of which we are able to judge: the book is remarkably clear and intelligible; its free and masculine style does Mr. Percival infinite credit.—Ed. *Med. Quart. Rev.*]

### SMALL HOSPITALS.

There are only fifteen beds in the hospital of Goettingen, and I do not wish for more. I do not think that the experienced practitioner is formed by the number of patients. Experience is the result, not of seeing merely, but of reflecting. It is not eating, but digestion, that gives strength. A physician who tells us that he visits a hundred and fifty, and even a greater number of sick people daily, has, in my opinion, so little pretensions to the title of an experienced man, that I would even deny that he had any experience at all.—*Lancet*, from *Richter's Med. and Surg. Observ.*

[Fifteen beds may be enough for the physician, but more, we think, are required for the pupils; just as a few books are enough to study and digest, but we wish for a library to consult: for the pupil is disappointed, if, in a year's attendance, he has not had an opportunity of witnessing almost everything that is remarkable in disease, and brilliant in operations.—Ed. *Med. Quart. Rev.*]

### PURE TANNIN.

Still more recently a friend of mine, M. Pelouse, professor at the Polytechnic School, whilst treating by æther, in a vertical tube, the powdered poison-nut (*Strychnos nux vomica*), made a discovery of much importance in organic chemistry, and I doubt not also in medicine. I allude to *tannin*. This immediate principle, so universal in the vegetable kingdom in the wood, the roots, and bark of the oak, the bark-tree, the ratanhia, &c.—which forms the active principle of almost all the astringents used in medicine,—this immediate principle had long since been obtained, but always impure, constantly combined with the acids or alkalies employed in its preparation, constantly insoluble and modified. But, in treating by æther the powder of St. Ignatius's bean, of the gall-nut, of catechu, and several other substances, in a vertical apparatus, M. Pelouse saw a yellowish liquid, transparent, and of a syrupous consistency, composed of tannin and æther, pass off: it was soon followed by a stream of pure æther, which remained floating on the surface of the dissolution of tannin. The æther carefully poured off, the syrupous dissolution was evaporated at a low heat, and gave a residue of tannin perfectly pure, crystallisable, soluble in water and alcohol; in short, possessing all the properties which characterize it in the plants in which it exists.

This simple process is perfectly applicable to the gall-nut, and yields a large proportion of tannin. This new produce thus prepared, will bear to all other astringents used in medicine, the same relation that quinine bears to bark, morphine to opium; and like them may, it appears to me, be substituted with advantage to its parent substances. To M. Pelouse it belongs to indicate the full bearing of his brilliant discovery, and I am happy to announce that he is on the point of publishing the results of his experiments on this interesting subject.—*The Monthly Journal of Medico-Chirurgical Knowledge.*

[This extract is from the polyglot Journal mentioned in our last number: in spite of its dog-English, its curiously small type, and its being three or four months behindhand, our contemporary (if it is not an anachronism to call it by that name,) has great merits: its articles are pregnant with good sense and practical information, and the plates of topographical anatomy are not only extremely useful, but remarkably beautiful.—Ed. *Med. Quart. Rev.*]

### ON THE ACTION OF CHLORINE ON METALLIC IODIDES.

By A. T. Thomson, m.d., Professor of Materia Medica in the University of London.

*To Mr. Richard Phillips.*

*5th May,* 1834.

Dear Sir: It has been long known that liquid chlorine, that is, a solution of chlorine in water, added to solutions of metallic iodides, or, as they then become, hydriodates, sets free the iodine, and thus enables it to be detected in minute quantity on the addition of starch. But it is also well known, that a very small excess of the solution of chlorine destroys the colour of the iodine of amidine, and renders the test fallacious. To remedy this disadvantage, I have substituted chlorine gas for the liquid chlorine, and find that it is capable of discovering the minutest portion of any hydriodate in solution, even in mixed fluids. The method of testing is to mix a small quantity of solution of starch in the fluid to be tested, and pouring on the surface of the liquid some chlorine gas; as soon as the gas reaches the surface a thin film of blue appears, and gradually pervades the whole of the liquid, if any hydriodate be present. The advantage of the test is the impossibility of adding too much, as the action commences on the surface, and the superabundant chlorine, which is mixed with the common air in the upper part of the test tube or the glass, is soon dissipated.

As a proof of the delicacy of the test, I may add, that four minims of a solution of hydriodate of potassa, containing one drachm in the fluid ounce, were added to a fluid ounce of water, and tested by the method above described, when the presence of free iodine was immediately rendered evident. The proportion of the hydriodate in this case being only $\frac{1}{900}$th, I conceive that the test is adequate for any experiment in which it may be required to ascertain the presence of a hydriodate in solution.

The theory of the process is too obvious to require any comment. Your making this test generally known through the medium of the Philosophical Magazine, will greatly oblige,

Yours faithfully,

A. T. Thomson.

—*The London and Edinburgh Philosophical Magazine.*

